# Considerations for Applying Route-to-Route Extrapolation to Assess the Safety of Oral Exposure to Substances

**DOI:** 10.3390/biom13010005

**Published:** 2022-12-20

**Authors:** Shruti V. Kabadi, Jeffrey Fisher, Benjamin Hung, Jason Aungst

**Affiliations:** 1 U.S. Food and Drug Administration, Center for Food Safety and Applied Nutrition, Office of Food Additive Safety, 5001 Campus Drive, HFS-275, College Park, MD 20740, USA; 2 U.S. Food and Drug Administration, National Center for Toxicological Research, 3900 NCTR Road, Jefferson, AR 72079, USA

**Keywords:** route-to-route, inhalation data, equivalent oral daily dose, toxicokinetics, volatile compounds, wash in–wash out

## Abstract

The safety evaluation of oral exposure to substances, such as food ingredients, additives, and their constituents, relies primarily on a careful evaluation and analysis of data from oral toxicity studies. When relevant oral toxicity studies are unavailable or may have significant data gaps that make them inadequate for use in safety evaluations, data from non-oral toxicity studies in animals, such as studies on inhalation, dermal exposure, etc., might be used in support of or in place of oral toxicity studies through route-to-route (R-t-R) extrapolation. R-t-R extrapolation is applied on a case-by-case basis as it requires attention to and comparison of substance-specific toxicokinetic (TK) and toxicodynamic (TD) data for oral and non-oral exposure routes. This article provides a commentary on the utility of R-t-R extrapolation to assess the safety of oral exposure to substances, with an emphasis on the relevance of TK and systemic toxicity data.

## 1. Introduction

The toxicokinetic (TK) and toxicodynamic (TD) profiles of substances may vary based on the exposure route. If appropriately conducted, oral toxicity studies are available for substances, such as food ingredients, additives, and their constituents; the safety assessment of oral exposure to such substances is based on oral toxicity data. However, if adequate data from animal toxicity or epidemiological studies are not available or the available studies have significant data gaps, non-oral toxicity studies, such as studies based on inhalation, dermal exposure, etc., might be used in support of or in place of oral toxicity studies through route-to-route (R-t-R) extrapolation [1,2,3]. R-t-R extrapolation is based on the application of an equivalent internal rather than external dose to assess the safety of substances [1,2,3,4]. Prior to applying R-t-R extrapolation, it is important to evaluate the TK and TD profiles of a substance for the relevant exposure routes. Because R-t-R extrapolation involves consideration of the relevant substance-specific TK and TD data, this approach should be applied on a case-by-case basis. In general, for some substances that demonstrate systemic biomarkers of toxicity, such as volatile compounds that readily enter the systemic circulation after exposure via a non-oral route (e.g., inhalation), R-t-R can be used to estimate the internal equivalent dose for safety assessment [5]. In contrast, for some substances that exhibit marked differences in TK and TD profiles between the relevant exposure routes or if the observed toxicity is based on the portal of entry, R-t-R extrapolation is not a viable alternative for safety assessment [6].

This paper is not intended for use as a guidance document for the application of R-t-R extrapolation to evaluate the safety of oral exposure to such substances. Instead, this paper provides a commentary on the considerations necessary to help ensure the appropriate use of R-t-R extrapolation to contribute to the safety assessment of oral exposure to substances when adequate oral toxicity data are not available.

## 2. R-t-R Extrapolation: Background and Concepts

**Figure 1:**
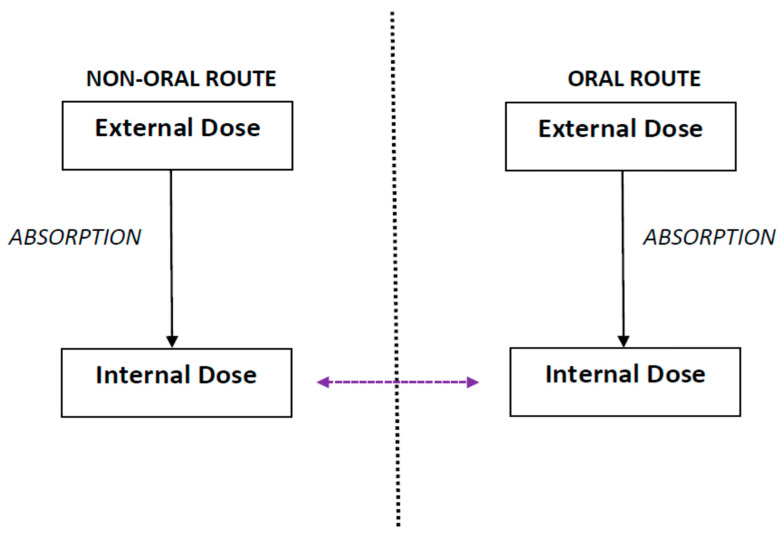
Route-to-route extrapolation refers to extrapolation of the internal dose from one exposure route, such as non-oral (inhalation, dermal, etc.) to another, such as oral, or vice-versa, such that prediction of effects is based on the internal dose instead of the external exposure.

R-t-R extrapolation is defined as the extrapolation of the internal dose from one exposure route to another exposure route such that prediction of effects is based on the internal dose instead of the external dose (Figure 1) [3]. The term “external dose” refers to the dose administered in the study, whereas the term “internal dose” refers to a fraction of the administered dose or the concentration of the administered substances that is absorbed and distributed in the body through systemic circulation [7]. R-t-R extrapolation predicts the equivalent dose and dosing regimen that produces the same observed effect as that obtained for a particular dose and dosing regimen by an alternate route [1,2,4].

Gerrity and Henry published a report [3] on the principles of R-t-R extrapolation based on the discussions from a workshop attended by several experts in Hilton Head, South Carolina, and Durham, North Carolina, in 1990. The United States Environmental Protection Agency (US EPA) [8] and the Organisation for Economic Cooperation and Development (OECD) [9] have published guidance documents that discuss the significance and relevance of exposure route for data-derived extrapolation to perform risk assessment. Over the past few years, several articles on the importance and application of R-t-R extrapolation to evaluate different toxicity endpoints have been published [4,5,10]. Recently, the Center for Food Safety and Applied Nutrition (CFSAN) of the United States Food and Drug Administration (US FDA) partnered with the Society of Toxicology (SOT) to organize a colloquium on “Route-to-Route Extrapolation in the 21st Century” that included presentations by leading experts in the field of R-t-R extrapolation and a stimulating discussion session on the diverse applications of R-t-R to address different aspects of exposure and safety assessments [11]. At present, there is no guidance document that specifically addresses the feasibility of performing R-t-R extrapolation to evaluate the safety of oral exposure to substances based on data from non-oral exposure studies when adequate oral toxicity data are not available.

The application of the R-t-R approach to evaluate the safety of oral exposure to substances assumes that data from studies on an alternate route of exposure, i.e., non-oral routes such as inhalation, etc., are appropriate to evaluate the safety of a substance after exposure via the route of interest, i.e., oral [3]. However, prior to making this assumption and applying R-t-R extrapolation, it important to examine and compare the TK and TD profiles of the substance between the evaluated exposure routes.

## 3. Assessing the Relevance of Available Toxicokinectic and Toxicodynamic Data for R-t-R Extrapolation

Assessing the relevance of available data from non-oral studies prior to applying R-t-R extrapolation to evaluate the safety of oral exposure to a substance requires consideration of the TK and TD profiles of the substance between the relevant routes of exposure, as discussed below:

i. Assessing TK equivalence by comparing TK profiles of the substance between oral and non-oral routes:

Assessment of TK equivalence includes comparison of internal exposure estimates or profiles of a substance between the two exposure routes [12]. Based on the availability of TK data on a substance upon exposure via both routes, this determination could be quantitative by estimation of TK parameters, such as area under the curve (AUC), bioavailability (F%), maximum concentration (Cmax), elimination half-life (t_1/2_), clearance, etc., [13] or semi-quantitative by evaluation of the overall absorption, distribution, metabolism, and elimination profiles without necessarily calculating any quantitative parameters (Table 1). If no TK data are available to assess the TK equivalence between the two exposure routes, some additional factors may be considered to predict TK parameters, particularly those related to the absorption and distribution profiles of the substance, as long as its chemical structure is well-characterized. These include physicochemical characteristics, such as molecular weight, n-octanol-water partition coefficient (log Kow), acid dissociation constant (pKa), vapor pressure, water or lipid solubility, etc. [14].

The TK of a substance may not always directly correlate with the observed effects. However, when toxicity is driven by a specific parameter, such as Cmax or AUC, such a parameter(s) could be helpful for establishing points of departure, such as no observed adverse effect levels (NOAELs) or low observed adverse effect levels (LOAELs), as measures of internal exposure. These data enable the assessment of internal exposure in conjunction with the administered doses and their relationship to the time course of the observed effects [15].

ii. Determining toxicological relevance by comparing the TD profiles of exposure to the substance between oral and non-oral routes:

Determination of toxicological relevance includes reviewing available toxicological data to compare the TD profiles of a substance between the two exposure routes. The following questions should be answered to determine the toxicological relevance of the reported toxic effects after non-oral exposure to a substance to those reported or predicted after its oral exposure:

1. Are the reported toxic effects after non-oral exposure only localized due to contact (i.e., related to its portal of entry) or a result of systemic exposure?
2. Are there differences in types and severity of toxic effects between the two exposure routes?
3. Are there any differences in known mechanisms of action that are specific to the different routes of exposure associated with the reported toxic effects?

If reported toxic effects after exposure to a substance via a non-oral or oral route are related to its portal of entry, such as localized irritation on the skin upon dermal exposure, in the lungs upon inhalation exposure, or in the gastrointestinal tract upon oral exposure instead of signs of systemic toxicity, such data cannot be used to apply R-t-R extrapolation to evaluate the safety of exposure to the substance. In contrast, if the reported toxic effects after non-oral exposure are due to systemic exposure, the types and severity of toxic effects, as well as any underlying known mechanisms of actions, should be compared. The purpose of this comparison is to identify the key differences and similarities in TD profiles between the two exposure routes to assess the toxicological relevance of reported effects after non-oral exposure to a substance to expected or reported effects after oral exposure.

## 4. Inhalation and R-t-R Extrapolation for Assessment of Oral Exposure to Substances

The method of converting an estimate based on a non-oral route of exposure, such as inhalation, to a corresponding estimate based on an oral route of exposure varies with the type of substance being evaluated. In general, the conversion method is based on the principles of inhalation dosimetry that support estimation of the internal dose of a substance upon inhalation exposure. These principles have been previously discussed by the US EPA [16] for derivation of an inhalation reference concentration (RfC), a dose-response estimate of a continuous inhalation exposure to the human population that is unlikely to have an appreciable risk of deleterious non-cancer health effects during a lifetime. In the context of assessing the safety of oral exposure to substances, such as food ingredients, additives, and their constituents, the exposure is expressed as an equivalent oral daily dose (mg/kg bw/d). In general, R-t-R extrapolation can be reliably applied to estimate an equivalent oral daily dose (expressed as mg/kg bw/d) from the corresponding inhalation exposure value (expressed as parts per million (ppm)) for volatile compounds (e.g., organic solvents such as styrene (CASRN: 100-42-5) [12] and isopropylbenzene (CASRN: 98-82-8)). Such compounds generally have a high vapor pressure, increasing their likelihood of entering systemic circulation upon inhalation.

It is important to incorporate any species-specific physiological differences if the relevant data are available to apply R-t-R extrapolation. Physiological parameters of processes, such as alveolar ventilation, which is critical for inhalation exposure, vary with the species. Alveolar ventilation is the process of the entrance of inspired air into the alveoli upon inhalation. Different species inhale different amounts of compounds upon inhalation exposure depending on their body size and level of cardiac exertion. Therefore, the rate of alveolar ventilation varies with species. AVR, which is expressed as ml/min/kg bw, is defined as the rate of alveolar ventilation per unit time. AVR estimates for specific strains are sometimes reported in published articles. However, when the AVR values are not reported, average values of AVR in rats (529 mL/min/kg bw), mice (1160 mL/min/kg bw), and humans (50 mL/min/kg bw) established by Brown et al. [17] can be used to estimate the equivalent oral daily dose.

If relevant TK data are available to predict the bioavailability after inhalation exposure, it is important to incorporate that estimate to apply R-t-R extrapolation. If TK studies indicate a rapid absorption profile of a substance after inhalation (e.g., a substance that has a high absorption coefficient or fast clearance from the lungs with a low probability of retention in the lungs), it can be assumed to be 100% absorbed into the systemic circulation. For example, N,N-dimethylformamide (CASRN: 68-12-2) is rapidly and readily absorbed into the systemic circulation upon inhalation exposure in rats and mice [18], as well as monkeys [19,20], such that its extent of absorption can be assumed to be 100% after inhalation exposure in these species. However, if a substance is not completely absorbed after inhalation exposure, it is critical to factor the extent of absorption into the conversion to apply R-t-R to evaluate its safety after oral exposure. For example, published studies indicate that styrene is 70% absorbed after inhalation exposure, whereas its absorption is almost 100% after oral exposure [21]. However, considering that the overall TK and TD profiles of styrene do not significantly differ between oral and inhalation exposure routes, it is appropriate to apply R-t-R based on inhalation data to evaluate the safety of styrene after oral exposure, provided that the conversion accounts for 70% of the exposed styrene being absorbed systemically after inhalation exposure [12].

When evaluating the relevance of data from inhalation studies, it is important to note that R-t-R extrapolation cannot be applied to the safety assessment of oral exposure for all substances. For substances for which the TK and/or TD profiles differ markedly between the evaluated exposure routes, R-t-R is not a viable option to evaluate their safety. For example, there are significant differences in disposition of cobalt (II, III) oxide after exposure via oral versus inhalation routes. Cobalt (II, III) oxide is not readily absorbed after inhalation exposure. The mean half-life (t_1/2_) of cobalt (II, III) oxide after inhalation exposure was reported to be 150–250 days in humans [22], whereas an oral exposure study indicated that cobalt (II, III) oxide was completely cleared within ten days after oral exposure in humans [23]. The longer t_1/2_ of cobalt (II, III) oxide after inhalation exposure is likely a result of retention of cobalt particles in the lungs due to slow translocation and mechanical clearance of these particles through the lungs. The rates of clearance of these particles through the lungs may also vary with their particle size [22]. Such factors associated with the movement and clearance of particles in the lungs are not relevant to the oral exposure route. The elimination of cobalt (II, III) oxide after inhalation exposure to humans occurred through both urine and feces, whereas its elimination after oral exposure occurred primarily through the urine. As a result of these TK differences of cobalt (II, III) oxide between the oral and inhalation routes, the available inhalation toxicity data are not relevant to assess its safety after oral exposure. Furthermore, R-t-R extrapolation cannot be applied to the safety assessment of oral exposure to substances such as certain water-soluble vapors, the inhalation exposures of which demonstrate a “wash in–wash out” effect [24]. The “wash in–wash out” effect represents a scenario in which most of the concentration of a substance administered via inhalation is exhaled, resulting in a limited opportunity for systemic absorption. In such cases, the concentration of the substance in the system is not an accurate representation of the systemic exposure to the substance when administered via the oral route because most of it is eliminated via exhalation prior to being absorbed.

## 5. Conclusions

Prior to applying R-t-R extrapolation to evaluate the safety of a substance, it is important to carefully examine and compare its TK and TD profiles between the two exposure routes. R-t-R extrapolation can be more reliably and accurately applied for some substances, such as volatile organic solvents, than others, the TK and TD profiles of which vary with their physiochemical properties and/or are related to the portal of entry. Therefore, the application of R-t-R extrapolation should be considered on a case-by-case basis to contribute to the safety assessment of substances after oral exposure.

## Figures and Tables

**Table 1:** Examining and comparing TK profiles of a substance between the evaluated exposure routes based on the availability of relevant TK data.

Availability of TK Data for the Evaluated Exposure Routes	Type of Assessment	Outcome
Adequate TK data are available with appropriate details of the studies (e.g., concentration vs. time curves, design and methods of the studies, assumptions/parameters of the models, etc.)	Quantitative	TK parameters such as area under the curve (AUC), bioavailability (%F), maximum concentration (Cmax), elimination half-life (t_1/2_), clearance, etc., for comparison between the two exposure routes.
Adequate TK data are available; however, pertinent details of the studies are missing (e.g., concentration vs. time curves are reported; however, information on the design, methods, dosing parameters, and/or data analysis are lacking.)	Semi-quantitative	Prediction of whether a substance is readily absorbed or rapidly eliminated by visual inspection of the curves and related details for the two exposure routes (AUC may be estimated as a marker of internal exposure, depending on the quality of the published curves.)
No TK data are available; however, the chemical structure of the substance is well-characterized.	Semi-quantitative	Prediction and comparison of TK profiles between the two exposure routes based on physicochemical properties, such as molecular weight, log Kow, pKa, vapor pressure, water, or lipid solubility.

## Data Availability

Not applicable.
